# Use of Antenatal Services in Kampung District, Cambodia

**DOI:** 10.1100/tsw.2003.95

**Published:** 2003-11-03

**Authors:** Ahmed A. Zafar, John E. Ehiri, Ebere C. Anyanwu

**Affiliations:** Technical Support Service, Aga Khan Health Service Pakistan, Mountain Inn Chitral, Pakistan

**Keywords:** maternal health, antenatal care, prenatal care, safe-motherhood, quality of care, Cambodia, developing countries

## Abstract

This study was conducted to assess factors that influence use of antenatal care services with both quantitative and qualitative designs. Methods used were structured questionnaire interviews and focus group discussions in the Kampung District, Kampot Province in Cambodia with a volunteer sample of 260 postnatal mothers. The outcome measure was factors influencing use of antenatal care services. The results showed that first-time mothers (primigravidas) were more likely to use antenatal services than multiparous mothers (OR = 1.87; p = 0.001). Mothers with some school education used antenatal services more than those with no school education (OR = 2.0; p = 0.01). Mothers engaged in professional occupations by virtue of their higher levels of educational attainment were more likely to use antenatal services than those engaged in agriculture (OR = 2.54; p = 0.001). Use of antenatal care services was higher in the districts whose health centers were supported by a foreign nongovernmental organization as compared to other districts with no such support (OR = 2.44; p = 0.001). Although services were generally inadequate, those that existed were underutilized by the mothers. Major factors influencing use of services include distance, lack of transport, and lack of awareness of the benefits of antenatal care by the mothers, thus resulting in a general notion that antenatal care is only important when problems occur during pregnancy. It is concluded that for remote villages, mobile antenatal clinics should be provided to improve access, and greater emphasis should be placed on health educating the mothers about the potential benefits of antenatal care, with special attention to multiparous mothers and those from the lower socio-economic class, among whom use of antenatal services was lowest.

## INTRODUCTION

Cambodia covers a total area of 181.040 sq km, with a population of 11 million[1]. The population growth rate is 2.3%, the fertility rate is 4.6% (1995–2000), and the population is expected to increase to 16.5 million by 2025[1]. Nearly half of the population (43.9%) is under 15 years of age, and illiteracy rates are high, particularly for women (21% for males, 45% for females). About 75% of the total work force is occupied in the agricultural sector, rice cultivation being the primary activity. As a result of conflicts, approximately 80% of the country's infrastructures, including health facilities, have been partly or completely destroyed[1]. On the 2001 United Nation’s Human Development Index[2], which measures a country’s achievements in terms of life expectancy, educational attainment, and adjusted real income, Cambodia is rated among the poorest. On a table of 162 countries, Cambodia ranks 121. With an annual Gross National Product (GNP) per capita of US$1,246, Cambodia is one of the poorest countries in Southeast Asia[1]. Both the country’s past and recent civil wars have had serious negative impact on the social and political environment, and have created a huge burden for the population, particularly women[1]. Only 53% of the population has access to basic health care, and since over 80% of the population live in the rural areas, efforts to provide health and basic social services to the population face not only financial, but also geographical and infrastructural challenges. Although women have limited access to education and health services, they comprise 65% of the labor force[1].

The population census of 1998, the first census in Cambodia in 30 years, enumerated a total of 5.92 million women and 5.51 million men in the country or 93 men for 100 women[3]. In some age groups, such as 40–44 years, there are only 65 men for 100 women. This demographic imbalance is a symptom of prolonged conflict and socio-political disintegration in the country[4], and women bear the greatest burden arising from this. As a consequence, many of them are widowed and/or single household heads[1]. The maternal mortality ratio of 500 per 100,000 live births is one of the highest in Asia[5]. Access to antenatal care is limited.

In our study setting, the Kampot province, only about 25% of women receive antenatal care, and less than 10% have a second antenatal consultation[3]. A recent study shows that many Cambodian women are concerned about dying in childbirth[6]. Traditional health beliefs (including maternal health beliefs) abound, and hence the impact on health-seeking behavior. For example, illness may be caused by an imbalance between "hot" and "cold" (intrinsic properties of substances and conditions), or by interference from the spirit world. Remedies for these types of illness include ritual and a range of magico-religious articles (e.g., amulets). Health facilities are poorly equipped, especially in the rural communities. One study found that in some district hospitals, there was no stethoscope in the labor ward and in some cases in the antenatal clinics[7]. There was no 24-h coverage, and when the unit was closed, patients had to be referred to private clinics or to make arrangements for a 3- to 4-h journey to the provincial hospital[7]. A baseline survey conducted in Cambodia found that 30% of the women who died during pregnancy or childbirth had received minimal antenatal care[8]. Another study of maternal mortality in the country found that the lifetime risk of maternal death is 0.024, meaning 1 out of 42 women who reached the age of 15 years and married, would die of pregnancy related causes[9]. According to the WHO technical working group on antenatal care[10], antenatal care should address both the psychosocial and medical needs of the woman, within the context of the healthcare delivery system and the culture in which she lives. Periodic health check-ups during the antenatal period are necessary to establish confidence between the woman and her healthcare provider, to individualize health promotional messages, and to identify and manage any maternal complications or risk factors. Antenatal visits are also used to provide essential services that are recommended for all pregnant women, such as tetanus toxoid immunization and the prevention of anemia through nutrition education and provision of iron/folic acid tablets[10]. Owing to long periods of deterioration in Cambodia’s health infrastructure and the inaccessibility of the few facilities for villagers, people prefer to be treated at home by the traditional practitioners they know and trust, and up to 70% of women deliver their babies at home[11].

Thus, maternal health is a subject of considerable importance in Cambodia and this study was an effort to assess factors associated with the use of antenatal care services in the District of Kampung, in the Kampot province. The objective was to provide information that could be used to improve planning of antenatal care services in the province.

## METHODS

### Study Setting

The study was carried out in Kampung District, Kampot Province, Cambodia. This is a catchment area with a population of about 54,000[3]. There are a total of 52 villages in the district. The villages are served by only five primary healthcare centers, thus making travel to the centers a difficult task for those mothers who live on the outskirts of the district. The health centers provide basic health services, including treatment of minor ailments, immunizations, basic biomedical examinations, weighing, and health education**.** Only two health centers supported by a Dutch nongovernmental organization (NGO) provided comprehensive obstetric care according to guidelines of the WHO[10].

### Sampling Technique

The sample frame consisted of all mothers who delivered in the district between June and September 2000. The mothers were identified through health facility sources (health center records), community sources (snowball technique —mothers identified through health center records were asked to identify persons they knew who delivered a baby within the study time period), and reports of village health workers and traditional birth attendants (TBAs). A total of 420 postnatal qualifying mothers were identified in the district. All 420 qualifying mothers were approached, and their consent to participate was solicited. The objective of the study was explained to the mothers in the local language (Khmer) by trained local research assistants before soliciting their voluntary participation in the study. The mothers were informed that their refusal to participate in the study would not result in the loss of any healthcare benefits from either the government or the foreign NGO. They were also told that they were free to withdraw from the study at any time they wished to do so. Of the 420 solicited, 260 mothers volunteered to participate in the study, giving a response rate of 62%.

### Data Collection

Quantitative data relating to factors that influence use of antenatal care were collected by means of a structured interviewer-administered questionnaire survey of all 260 participants. Further qualitative study to obtain in-depth views of the mothers regarding access to antenatal care was undertaken by means of six focus group discussion sessions involving 60 (23%) of the 260 mothers randomly selected from the study sample. Basic descriptive data collected by means of the structured questionnaire included educational attainment, parity, outcomes of last pregnancy and delivery. Issues covered in the focus group sessions included perceived importance of antenatal care and factors that hinder mothers’ ability to benefit from available services.

### Data Analysis

Quantitative data arising from the questionnaire interviews were analyzed, using Epi-Info 2000[12]. The subject matter categories approach to content analysis[13] was used to analyze qualitative data arising from the focus group discussion sessions. Content analysis is a research tool used to analyze the presence of certain words or concepts within texts or sets of texts. Using content analysis, researchers quantify and analyze the presence, context, meanings, and relationships of such words and concepts, then make inferences about the messages. To conduct a content analysis of the focus group discussion sessions, the transcripts were coded and broken down into manageable categories on a variety of levels — word, word sense, phrase, sentence, or theme/subject matter — and then examined using the conceptual analysis method. The groups constituted the units of analysis, and the analysis was undertaken in a group-by-group progression. In conducting the analyses, the first author and the local research assistant independently examined data generated by the first two focus group sessions, and based on these, developed coding schemes that were compared, validated, and applied to the remainder of the group sessions.

## RESULTS

### Characteristics of Participants

The characteristics of the study participants are shown in Table 1. The mean age of the mothers was 28 years, ranging from 15–44 years; 24% (n = 63) were first-time mothers, while 76% (n = 197) were multigravidas. Slightly more than half (57%; n = 147) delivered at a health facility, the remaining 43% delivered at home. A majority (65%; n = 169) was married, a few were single (5%; n = 12), and widowed (30%; n = 79). Of educational level attained, 71 (27%) had no formal school education, 120 (46%) had primary school education, while 60 (23%) had secondary education. A few (4%; n = 9) had postsecondary school education, and most were homemakers or engaged in agriculture. Slightly more than half (52%; n = 135) used the antenatal care during their last pregnancy.

### Adverse Obstetric History

Many of the mothers (50%; n = 131) have had adverse obstetric history. Of those, 75% indicated that they had a serious health problem requiring hospitalization during their last pregnancy. The most common health problems cited by the mothers were anemia (40%; n = 53), hypertensive disorder (including pre-eclampsia and eclampsia [18%; n = 23]), vaginal bleeding (6%; n = 8), and malaria (36%; n = 47).

### Reasons for Seeking Antenatal Care

Of the 260 mothers surveyed, only 135 (52%) admitted using available antenatal health services during their last pregnancy, the remaining 125 (48%) did not. Most mothers indicated that they attended the health centers mainly to get treatment when they were sick (81%; n = 109); specifically to obtain tetanus toxoid immunization (16%; n = 22), and to have the position of their baby checked (3%; n = 4). In both the questionnaire and focus group discussion sessions, mothers expressed difficulty with distance, travel cost, and availability of transport. During the focus group discussions, many of the mothers who used antenatal services expressed the belief that

**TABLE 1**
**Characteristics of the Respondents**

| Parameter | N = 260; No. (%) |
|---|---|
| **Age of mother** | |
| 15–19 | 18 (7) |
| 20–24 | 63 (24) |
| 25–29 | 65 (25) |
| 30–34 | 62 (24) |
| 35+ | 52 (20) |
| **Parity** | |
| Primigravida | 63 (24) |
| Multigravida | 197 (76) |
| **Marital status** | |
| Married | 169 (65) |
| Single | 12 (5) |
| Widowed | 79 (30) |
| **Mothers' education** | |
| No school education | 71 (27) |
| Primary school | 129 (50) |
| Secondary school | 50 (19) |
| Postsecondary school | 10 (4) |
| **Mothers' occupation** | |
| Homemaker | 89 (34) |
| Agriculture | 95 (37) |
| Trader | 29 (11) |
| Office professional | 47 (18) |
| **Husband's education** | |
| No school education | 31 (12) |
| Primary school | 122 (47) |
| Secondary school | 72 (28) |
| Postsecondary school | 35 (13) |
| **Husband's occupation** | |
| Agriculture | 228 (87) |
| Trader | 15 (6) |
| Office professional | 17 (7) |
| **Have had adverse obstetric history** | |
| Yes | 96 (37) |
| No | 164 (63) |
| **Adverse obstetric history** | |
| Stillbirth | 8 (3) |
| Serious illness requiring hospitalization during last pregnancy | 76 (29) |
| Death of a child under 5 years of age | 8 (4) |
| Death of two or more children under 5 years of age | 4 (2) |
| **Used antenatal care during last pregnancy** | |
| Yes | 135 (52) |
| No | 125 (48) |

antenatal care was good for the health of the mother and the baby. One of the women, who had already had many children, said she was afraid during her last pregnancy because she was constantly unwell and unusually weak. She thought she might have problems during delivery and hence she visited the health center four to five times for antenatal consultations.

## User Fees

Most of the nonusers of antenatal care revealed a number of factors that hindered them from using antenatal services. Prominent among these were the fact they had no problem during pregnancy (76%; n = 95) and hence did not see need to seek antenatal care, had difficulty with transportation (14%; n = 17), did not know the location of antenatal care facilities (6%; n = 7), and cost (5%; n = 6). It was revealed that a mother was required to pay 2,000 Riel (1 US$ = 3,850 Riel) for antenatal care in addition to transportation costs. Most of the women were of the opinion that the fees should be abolished or significantly reduced. One of the women commented, "the payment of 5,000 Riel for transport in addition to 2,000 Riel for antenatal care is beyond the reach of many of us". Some of them commented that they sometimes forgot to go for antenatal care, because they were very busy working to get money to support their families. Only very few mothers (2%; n = 3) complained about negative attitude of health workers.

## Parity

As shown in Table 2, primigravidas used antenatal care more often than multigravidas. The odds of using antenatal care were 1.87 times higher among primiparous mothers than among those who were multiparous (OR = 1.87; $p$ = 0.001). Multiparous mothers were also less likely to have received TT vaccination during their antenatal period than primiparous mothers (OR = 2.10; $p$ = 0.001). In general, women who used antenatal care services had 16.79 times a better chance of being inoculated with TT vaccine (OR = 16.79; $p$ = 0.001).

## Age

Age of the mother was not a significant predictor of utilization of antenatal care in this study ($p$ = 2.19), since there was no marked difference between mean age of users (28 years) and nonusers (29 years) of antenatal care services.

## Waiting Time

Waiting time to see health personnel during an antenatal visit was not cited as an important factor in the use of antenatal services in the district. As indicated by the mothers, average waiting time to see health personnel during a visit to a health center was 15 min (ranging from 10 min to 1 h).

## Ability to Pay for Antenatal Services

When asked whether user fees were a factor in nonuse of antenatal services, some (26%; n = 68) admitted that they could not pay for the services. Those who expressed the ability to pay were asked to indicate how much they would be willing to pay to obtain the services that they need; 91 (35%) said they would pay up to 1,000 Riel, 52 (20%) would pay 1,100–2,000 Riel, 49 (19%) thought that they would pay 2,001–5,000 Riel for antenatal care.

**TABLE 2**
**Factors Influencing Use of Antenatal Care**

| Parameter | Used Antenatal Services — N = 135; No. (%) | Did Not Use Antenatal Services — N = 125; No. (%) | Odds Ratio | *p*-Values |
|---|---|---|---|---|
| **Parity** | | | | |
| Primigravida | 40 (63) | 23 (37) | 1.87 | <0.001* |
| Multigravida | 95 (48) | 102 (52) | | |
| **Mothers' education** | | | | |
| School education | 107 (79) | 82 (66) | 1.19 | 0.11 |
| No school education | 28 (21) | 43 (34) | | |
| **Mothers' s occupation** | | | | |
| Professional/clerical/sales | 58 (58) | 42 (42) | 2.54 | <0.001* |
| Agricultural | 77 (48) | 83 (52) | | |
| **Husband's occupation** | | | | |
| Professional/clerical/trading | 24 (75) | 8 (25) | 3.16 | <0.001* |
| Agricultural | 111 (49) | 117 (51) | | |
| **Age of mother** | | | | |
| <29 | 80 (55) | 66 (45) | 1.30 | 0.3 |
| 30+ | 55 (48) | 59 (52) | | |
| **Adverse obstetric history** | | | | |
| Yes | 71 (53) | 25 (20) | 4.44 | <0.001* |
| No | 64 (47) | 100 (80) | | |
| **Foreign NGO support** | | | | |
| Yes | 67 (65) | 36 (35) | 2.44 | <0.001* |
| No | 68 (43) | 89 (57) | | |
| **Parity and use of TT vaccine** | | | | |
| Primigravida | 42 (31) | 21 (17) | 2.24 | <0.01* |
| Multigravida | 93 (69) | 104 (83) | | |
| **Antenatal care is important for every pregnant women** | | | | |
| Yes | 118 (87) | 71 (57) | 5.28 | <0.001* |
| No | 17 (13) | 54 (43) | | |

## Problems During Last Pregnancy and Use of Antenatal Services

Of the 135 mothers who admitted using antenatal care in their last pregnancy, 71 (53%) had previous adverse obstetric history as compared to only 25 (20%) of those who did not use antenatal services. This suggests that women who experienced adverse obstetric outcome in their last pregnancy were more likely to attend antenatal care clinic as compared to those who had no such experience (OR = 4.44; $p = 0.001$).

## DISCUSSION

One important function of antenatal care services is to identify and monitor services for health promotion and disease prevention among pregnant women. Antenatal care not only provides preventive interventions, but also nutrition, health education, and postpartum family planning[14]. While increasing the content and coverage of antenatal care is of critical importance in many less-developed countries, ensuring that mothers make optimal use of the limited available services is equally crucial. The WHO[11] recommends that women attend at least four antenatal visits as follows: first visit by the end of the fourth month (16 weeks) to screen for and treat anemia and syphilis, and to screen for risk factors and medical conditions that can best be addressed in early pregnancy, to initiate prophylaxis where required (e.g., anemia, malaria) and to commence the development of an individualized birth plan; second visit in the sixth or seventh month (24–28 weeks); third visit in the eight month (32 weeks) to screen for pre-eclampsia, multiple gestation, anemia, and to further develop the individualized birth plan, and fourth visit in the ninth month (36 weeks) to identify fetal lie/presentation and to update the individualized birth plan. The fact that most of our study participants did not attend antenatal at all throughout their last pregnancy is very worrying. Opportunities for detection and management of complications would have been lost, and hence the high levels of adverse outcomes reported by the women.

### Study Sample

The 260 mothers (out of 420) who participated in this study represent a response rate of 62%. In an effort to understand whether the 160 mothers who did not participate in the study were different from those who participated, we identified five of these in the community and interviewed them. Of these five, only one visited a health center for antenatal care during her last pregnancy. The remaining four visited traditional practitioners and delivered their babies at home. The socio-demographic characteristics and opinions expressed by the five nonparticipating mothers regarding antenatal services in the district were not markedly different from those expressed by the participating mothers. Thus, while the level of response in this study is a factor for consideration in interpreting the results, we do not have any reason to believe that the nonrespondents were different from the study participants, and results can be taken with confidence.

### Adequacy and Quality of Antenatal Care Services

This study examined the content and quality of antenatal care services provided to women through outreach activities in relation to standard guidelines for content of antenatal care[10] which emphasizes three components: assessment (history, physical examination, and laboratory tests), health promotion, and care provision. In two health centers, Kampung Trach and Kanthor, staff was supported by a foreign NGO that provided training, supervision, and regular supplies. Antenatal services provided by these centers were in line with WHO guidelines[10,14]. There was no such support in the other three health centers in the district. This support made a significant difference and has an impact on use of services as a majority of those who used antenatal services visited the NGO-supported facilities (OR = 2.44; $p$ = 0.001). Another important contributing factor could be the fact that these two NGO-supported facilities are located in the more urban areas of the district where the socio-economic status of the families (education, employment, and income) are significantly better than in the rural villages.

## Education

There is a positive association between level of education of the mother and use of antenatal care services. The tendency is that the higher the level of education of the woman, the greater the chance that she will use antenatal care services. Evidence from previous research suggests that maternal education is one of the strongest determinants of utilization of healthcare services[9,15,16,17]. Education enhances the autonomy of the mother so that she develops greater confidence and capacity to make decisions regarding her own health. Education also increases self-awareness and acceptance of modern medical practices[9,18].

## Occupation of Husband

There is a positive association between the occupation of the women's husband and the likelihood that she would receive antenatal care. Women with husbands who were office professionals were more likely to receive antenatal care than women whose husbands were agricultural workers. This may be due to the fact that because of the husband's education, and hence occupation, the family was more likely to live in an urban area, and so would have easier access to facilities. Similar findings emerged from studies in Ghana[19], Jordan[20], and Kenya[21] where women whose husbands are professionals were more likely to receive antenatal care than women whose husbands are laborers. A similar finding emerged in Cambodia in a National Health Survey by Ministry of Health[3], which found a positive relationship between husbands' occupation and use of antenatal care service by their wives. Household socio-economic status, particularly the employment status of the husband is important as it influences both the frequency and timing of antenatal care visits[3]. Studies in many parts of the world have demonstrated an association between lower socio-economic status and underutilization of health services[16]. Women whose husbands are professionals are usually of high socio-economic status, which empowers them to seek modern health services as they can afford to pay for antenatal care services and associated transportation cost.

## Location of Services

Results of this study show that distance is an important factor in the use of antenatal services. The factors identified were consistent with the previous studies, which report transportation problem and distance as factors that account for the differential use of health services[22]. There is a strong negative association between longer travel distance and travel time to the health facility and the number of times the facility is used. The cost of transport can be a strong disincentive to use of the health facility, and mothers expressed this issue during focus group discussions. Similar findings demonstrated in a study in Nepal where long distance and cost of travel to health facilities were a disincentive to utilization of antenatal care services[23].

## Waiting Time

A common belief is that excessive waiting time discourages health center use. It has been found in other studies that waiting time at an antenatal clinic has a negative impact on attendance[24]. However, our study did not demonstrate any such association. In the focus group discussions, many mothers indicated that they "have waited for up to 2 h for services". A majority did not think that this waiting time was excessive. It can thus be argued that what constitutes excessive waiting time is highly influenced by culture. Rural Cambodians simply expect to have to wait for government services and will do so patiently for periods of time that others would find intolerable[8]. A similar finding were observed in a study in the Philippines[25] where it was found that waiting time for a visit in both urban and rural areas is not a significant predictor of usage.

### Problems in Previous Pregnancy

Women who had an obstetric problem in a previous pregnancy were more likely to be concerned about antenatal care and therefore, more likely to use antenatal services for subsequent pregnancies than women who had no such experience. Similar findings have been reported in a study by Matthews and Diamond[22]. Where poverty is rife, uptake of preventive services tends to be low, especially where if user fees are charged[26]. It is therefore not surprising that many of mothers did not see need to go for antenatal care unless they had a specific health problem. Unfortunately, the antenatal services are best equipped to render maternal health promotion and preventive rather than curative or emergency services. This underscores the importance of health education to change mothers' perceptions of the rationale for antenatal care.

## CONCLUSION

Despite the availability of antenatal care services in Kampung District almost 48% of the women did not use the services. Although services were limited, those that do exist were still underutilized. Some of the key reasons for this underutilization were limited awareness of the rationale for antenatal care for pregnant women, and location of the services. Furthermore, women with higher parity and women with limited or no school education used antenatal care services less frequently. Proximity to antenatal care services was important and long distance to nearest antenatal care facility was an obstacle to use of services. The fact that the NGO-supported facilities were used more frequently than the other government-funded facilities suggested the need for increased supplies, support, and education of mothers on the benefits of antenatal care services. The provision of more accessible care through outreach clinics will also help to improve access to, and use of antenatal care in the district[27], but this would require additional resources, including trained personnel. For remote villages, mobile antenatal clinics should be provided to improve access, and greater emphasis should be placed on multiparous mothers and those from the lower socio-economic class, among whom use of antenatal services was lowest.

## BIOSKETCHES

**Ahmed A. Zafar** is Manager, Technical Support Service, Aga Khan Health Service, Mountain Inn, Chitral, Pakistan. Email: zafar.ahmed@akhsp.org and akzafar330@yahoo.com

**John E. Ehiri** is Assistant Professor, Department of Maternal and Child Health, School of Public Health, University of Alabama at Birmingham. E-mail: jehiri@uab.edu

**Ebere C. Anyanwu** is a Clinical Neurophysiologist and the Director of Cahers Neurosciences Research Inc., Houston Texas. Dr. Anyanwu is also a member of the Biomonitor Research Group, Department of Chemistry, Texas Southern University, and an Adjunct Professor of Anatomy and Physiology at the North Harris and Montgomery Colleges in Houston and The Woodlands, respectively. E-mail: ebereanyanwu@msn.com
